# Species-level resolution for the vaginal microbiota with short amplicons

**DOI:** 10.1128/msystems.01039-23

**Published:** 2024-01-26

**Authors:** Wei Qing, Yiya Shi, Rongdan Chen, Yin'ai Zou, Cancan Qi, Yingxuan Zhang, Zuyi Zhou, Shanshan Li, Yi Hou, Hongwei Zhou, Muxuan Chen

**Affiliations:** 1 Microbiome Medicine Center, Division of Laboratory Medicine, Zhujiang Hospital, Southern Medical University, Guangzhou, China; 2 Department of Medical Laboratory, The Central Hospital of Wuhan, Tongji Medical College, Huazhong University of Science and Technology, Wuhan, Hubei, China; 3 Department of Medical Laboratory, Shenzhen People’s Hospital, The Second Clinical Medical College of Jinan University, The First Affiliated Hospital of South University of Science and Technology, Shenzhen, Guangdong, China; University of Massachusetts Medical School, Brookline, Massachusetts, USA

**Keywords:** vaginal microbiota, 16S rRNA gene sequencing, taxonomic classification, species-level resolution

## Abstract

Specific bacterial species have been found to play important roles in human vagina. Achieving high species-level resolution is vital for analyzing vaginal microbiota data. However, contradictory conclusions were yielded from different methodological studies. More comprehensive evaluation is needed for determining an optimal pipeline for vaginal microbiota. Based on the sequences of vaginal bacterial species downloaded from NCBI, we conducted simulated amplification with various primer sets targeting different 16S regions as well as taxonomic classification on the amplicons applying different combinations of algorithms (BLAST+, VSEARCH, and Sklearn) and reference databases (Greengenes2, SILVA, and RDP). Vaginal swabs were collected from participants with different vaginal microecology to construct 16S full-length sequenced mock communities. Both computational and experimental amplifications were performed on the mock samples. Classification accuracy of each pipeline was determined. Microbial profiles were compared between the full-length and partial 16S sequencing samples. The optimal pipeline was further validated in a multicenter cohort against the PCR results of common STI pathogens. Pipeline V1–V3_Sklearn_Combined had the highest accuracy for classifying the amplicons generated from both the NCBI downloaded data (84.20% ± 2.39%) and the full-length sequencing data (95.65% ± 3.04%). Vaginal samples amplified and sequenced targeting the V1–V3 region but merely employing the forward reads (223 bp) and classified using the optimal pipeline, resembled the mock communities the most. The pipeline demonstrated high F1-scores for detecting STI pathogens within the validation cohort. We have determined an optimal pipeline to achieve high species-level resolution for vaginal microbiota with short amplicons, which will facilitate future studies.

**IMPORTANCE:**

For vaginal microbiota studies, diverse 16S rRNA gene regions were applied for amplification and sequencing, which affect the comparability between different studies as well as the species-level resolution of taxonomic classification. We conducted comprehensive evaluation on the methods which influence the accuracy for the taxonomic classification and established an optimal pipeline to achieve high species-level resolution for vaginal microbiota with short amplicons, which will facilitate future studies.

## INTRODUCTION

Compared with gut, human vagina has relatively less diverse microbial communities, which are commonly dominated by *Lactobacillus* in reproductive-age women and sometimes colonized by a collection of facultative and obligate anaerobes (1, 2). Accumulating studies have linked vaginal microbiota to host health outcomes including host inflammatory response (3), obesity (4), prenatal stress on offspring (5), preterm birth (6), HIV acquisition (7) and treatment (8), ovarian cancer (9), human papillomavirus (HPV) infection, and cervical intraepithelial neoplasia (CIN) (10, 11). Consequently, a variety of specific bacterial species were found to play vital roles in different host's physiological and pathological processes (3, 6–8, 10–13). Some vaginal microbes from the same genus but different species have been proven to exert completely opposite influences on vaginal niche, that is, *Lactobacillus crispatus* is a well-recognized probiotic for treating bacterial vaginosis (BV) (14) while *L. iners* has been identified as a potential intermediary associated with adverse vaginal health outcomes (10, 15, 16). Therefore, it is of vital importance to analyze the vaginal microbiota with species-level resolution.

For vaginal microbiota studies, diverse 16S rRNA gene regions were applied for amplification and sequencing (1, 6, 8, 17–20), which greatly affect the comparability between different studies as well as the species-level resolution of taxonomic classification (21). Despite the sequencing region of 16S rRNA gene, other factors can also influence the resolution of species-level classification. Bokulich et al. reported how the taxonomic classification method influenced the species-level resolution (22), while Van Der Pol et al. highlighted the importance of the reference database (23).

With the overwhelming data generated by vaginal microbiota researchers, some endeavors have been made to improve the species-level analysis of the vaginal microbiota (21, 23–25). However, despite there were inevitable limitations among these studies, contradictory conclusions have been yielded. Hence, to determine a pipeline for analyzing the vaginal microbiota with a high species-level resolution, we first conducted a computational evaluation on the sequences of ever reported vaginal bacterial species downloaded from NCBI. Through simulated amplification with various primer sets and taxonomic classification using different combinations of algorithms and reference databases. We determined the optimal pipeline to have the highest accuracy for classifying the vaginal species. Moreover, vaginal swabs were collected from participants with diverse vaginal microecology to construct the 16S full-length sequenced mock communities, with which computationally and experimental amplifications as well as sequencing were performed. Microbial profiles were compared between the full-length and the partial 16S sequencing data to further confirm the pipeline. Finally, the pipeline was implemented and validated in a multicenter cohort comprising 7,076 women in reproductive age across China, by comparing the results obtained from 16S sequencing with those obtained through PCR test for various sexually transmitted infection (STI) pathogens.

## MATERIALS AND METHODS

### Computational evaluation

From previous research on bacteria in human vagina (Table S1), 162 reported species were retrieved, of which nucleotide sequences with species-level annotation were downloaded from NCBI, including both 16S rDNA and whole genome sequences. We employed SeqKit tool suite (26) to filter out records with a length less than 200 bp and those that possess duplicate accession numbers or sequences, and simulate amplification with seven primer sets (Table 1) targeting V1–V2, V1–V3, V3–V4, V4, V4–V6, V5–V7, and V7–V9 regions of 16S rRNA gene, allowing appropriate mismatches. The amplicons were aligned using MAFFT (27). Masked alignments were applied for phylogeny construction using FastTree (28), which were visualized using iTOL (29).

**TABLE 1:** Primer sets for 16S rRNA gene amplification

Targeting regions	Primer name	Primer sequence
Full-length (30)	27F 1492R	5′-AGRGTTYGATYMTGGCTCAG-3′ 5′-RGYTACCTTGTTACGACTT-3′
V1–V2 (1, 31)	27F-A 27F-B 338R	5′-AGAGTTYGATYMTGGCTYAG-3′ 5′-AGARTTTGATCYTGGTTCAG-3′ 5′-TGCTGCCTCCCGTAGGAGT-3′
V1–V3 (32)	27F-A 27F-B 533R	5′-AGAGTTYGATYMTGGCTYAG-3′ 5′-AGARTTTGATCYTGGTTCAG-3′ 5′-ATTACCGCGGCTGCTGG-3′
V3–V4 (25)	338F 806R	5'-ACTCCTACGGGAGGCAGCAG-3′ 5'-GGACTACHVGGGTWTCTAAT-3′
V4 (23)	515F 806R	5′-GTGCCAGCMGCCGCGG-3′ 5′-GGACTACHVGGGTWTCTAAT-3′
V4–V6 (33)	533F 1100R	5′-GTGCCAGCMGCCGCGGTAA-3′ 5′-GGGTTGCGCTCGTTG-3
V5–V7 (34)	799F 1175R	5′-AACMGGATTAGATACCCKG-3′ 5′-ACGTCRTCCCCDCCTTCCT-3′
V7–V9 (35)	1115F 1492R	5′-YAACGAGCGCAACCC-3′ 5′-CGGTTACCTTGTTACGACTT-3′

Different reference databases (Greengenes2 Release 2022.10 [36], SILVA Release 138 [37], and RDP training set No. 18 [38]) and taxonomic classification methods (BLAST+ [39], VSEARCH [40], and Sklearn [41]) in QIIME2 (22) with the default settings to determine the optimal pipeline for classifying the known bacterial species sequences.

### Collection and processing of the vaginal swabs

Metadata and vaginal secretion swabs of the participants were withdrawn from our ongoing cohort. Participants recruitment and sample collection, processing and testing including HPV genotypes and other STI pathogens detection were described in a previous publication (42).

Indicators of vaginal discharge morphology and function were examined. Morphotype was defined by microscopy according to estimate count of cells (e.g., epithelial cell, red and white blood cell, pus cells, and clue cells) and pathogen (e.g., gram-positive cocci, gram-negative bacilli, spores, trichomonas, etc.). Hydrogen peroxide (H_2_O_2_) and leukocyte esterase (LE) activities were measured to evaluate the activity of dominant bacteria and the damage to vaginal mucosa, respectively. We evaluated the overgrowth of pathogenic bacteria by assessing the levels of sialidase (SNA), coagulase (COA), or β-glucuronidase (β-GD). On an automated vaginitis detection system, vaginal pH was measured using pH test paper. We calculated the Nugent score by assessing the relative number of bacterial morphotypes on gram-stained vaginal smears, which gave a score between 0 and 10. A normal score was <4, a moderate score was 4–6, and a score of ≥7 indicated bacterial vaginosis.

The vaginal secretions were eluted from each swab to a centrifuge tube by vertexing and centrifuging with saline solution. The vaginal eluents were evenly mixed and then delivered 500 µL per tube as mock community samples.

### DNA extraction, PCR amplification, and 16S sequencing

DNA was extracted using the QIAamp BiOstic Bacteremia DNA Kit (QIAGEN) following the manufacturer's instructions. For the mock samples performed 16S rRNA gene full-length sequencing, the bacterial 16S rRNA genes were amplified using the universal bacterial primers 27F and 1492R (Table 1). The PCR amplification was performed in triplicate as follows: initial denaturation at 95°C for 3 min, followed by 27 cycles of denaturing at 95°C for 30 s, annealing at 60°C for 30 s and extension at 72°C for 45 s, and single extension at 72°C for 10 min, and end at 4°C. Purified PCR products were pooled in equimolar and DNA library was constructed. Purified libraries were then sequenced on the Pacbio Sequel II System (Pacific Biosciences) by Majorbio Bio-Pharm Technology Co. Ltd. (Shanghai, China).

For the partial 16S rRNA gene sequencing, different hypervariable regions of the bacterial 16S rRNA gene were amplified using the primer sets in Table 1. For the mock samples sequenced targeting the V1–V2 and V1–V3 regions, amplification was performed by a mixture of primers 27F to maximize sequence type discovery and eliminate the PCR amplification bias (31). The 27F primer mixture was: fourfold 27F-A and onefold 27F-B. All samples were amplified in triplicate as follows: initial denaturation at 95°C for 5 min, followed by 29 cycles of denaturing at 94°C for 30 s, annealing at 52°C for 30 s and extension at 72°C for 1 min, single extension at 72°C for 10 min, and end at 4°C. Purified amplicons were pooled in equimolar amounts and paired-end sequenced on Illumina MiSeq PE300 platform and Novaseq PE250 platform (only for 10 samples that amplified the V1–V3 region).

### Sequencing data processing

PacBio raw reads were processed using the SMRTLink analysis software (Version 8.0) to obtain demultiplexed circular consensus sequence (CCS) reads with a minimum of three full passes and 99% sequence accuracy. CCS reads were barcode-identified and length-filtered, for which sequences that were either shorter than 1,000 bp or longer than 1,800 bp were removed. The optimized-CCS reads were denoised using the dada2 package in R software, following Callahan's pipeline (43), with the ASVs (amplicon sequence variants) table and representative sequences obtained. The representative sequences were aligned and annotated against the NCBI Nucleotide Sequence Database using BLAST (Version 2.11.0). Alignments were discarded if the e-value more than 10^−10^ and the identity less than 99%, and those with non-standard or vague species names were excluded either. Computational amplifications based on the full-length 16S rRNA gene representative sequences were performed using Seqkit with the same primer sets as in computational evaluation (Table 1).

The raw sequencing data generated from both Illumina Miseq and Novaseq were demultiplexed into sample pair-end fastq files based on unique barcodes and truncated by cutting off the barcodes and primer sequences using a customized Perl script. For the Illumina Miseq data, paired-end sequences were imported to QIIME2 (Version 2021.11) and were processed using the DADA2 (44) denoise-paired plugin for denoising. While forward sequences were denoised using the DADA2 denoise-single plugin (Version 2021.11) for the Illumina Novaseq data. The taxonomic classification was performed following the optimal pipeline for each 16S regions determined in computational evaluation.

The ASVs table for each 16S rRNA gene region was merged with the full-length ASVs table. Using the Vegan package (Version 2.6.4) in R software (Version 4.1.3), the distance matrix of each sample was calculated based on different methods including Bray Curits, Euclidean, Jacard, and Kulczynski. Furthermore, the principal coordinate analysis (PCoA) was performed using the Ape package (Version 5.6.2). Data visualizations and statistical analyses were performed using ggplot2 (Version 3.4.0).

### Calculation of the confusion matrix related metrics

Positive of a certain STI pathogen on a participant was defined as its relative abundance determined by our pipeline on 16S sequencing data more than or equal to 0.1%. Confusion matrix of our pipeline in classifying STI pathogens on 16S sequencing data against the PCR results was constructed, and its related metrics was calculated using R package caret. In the confusion matrix, true positive (TP) means the participants identified as positive by both 16S sequencing and PCR test, false positive (FP) means the participants identified as positive by 16S sequencing but negative by PCR test, true negative positive (TN) means the participants identified as negative by both 16S sequencing and PCR test, and false negative (FN) means the participants identified as negative by 16S sequencing but positive by PCR test.

The confusion matrix related metrics were calculated as the follows: sensitivity = TP/(TP + FN), specificity = TN/(TN + FP), precision = TP/(TP + FP), recall = TP/(TP + FN), F1-score = 2 × precision × recall/(precision + recall), accuracy = (TP + TN)/(TP + TN + FP + FN), Kappa = (accuracy – P_e_)/1 – P_e_ [Pe = (TP + FP)(TP + FN) + (FN + TN)(FP + TN)/(TP + TN + FP + FN)^2^].

## RESULTS

### Computational evaluation

**Fig 1:**
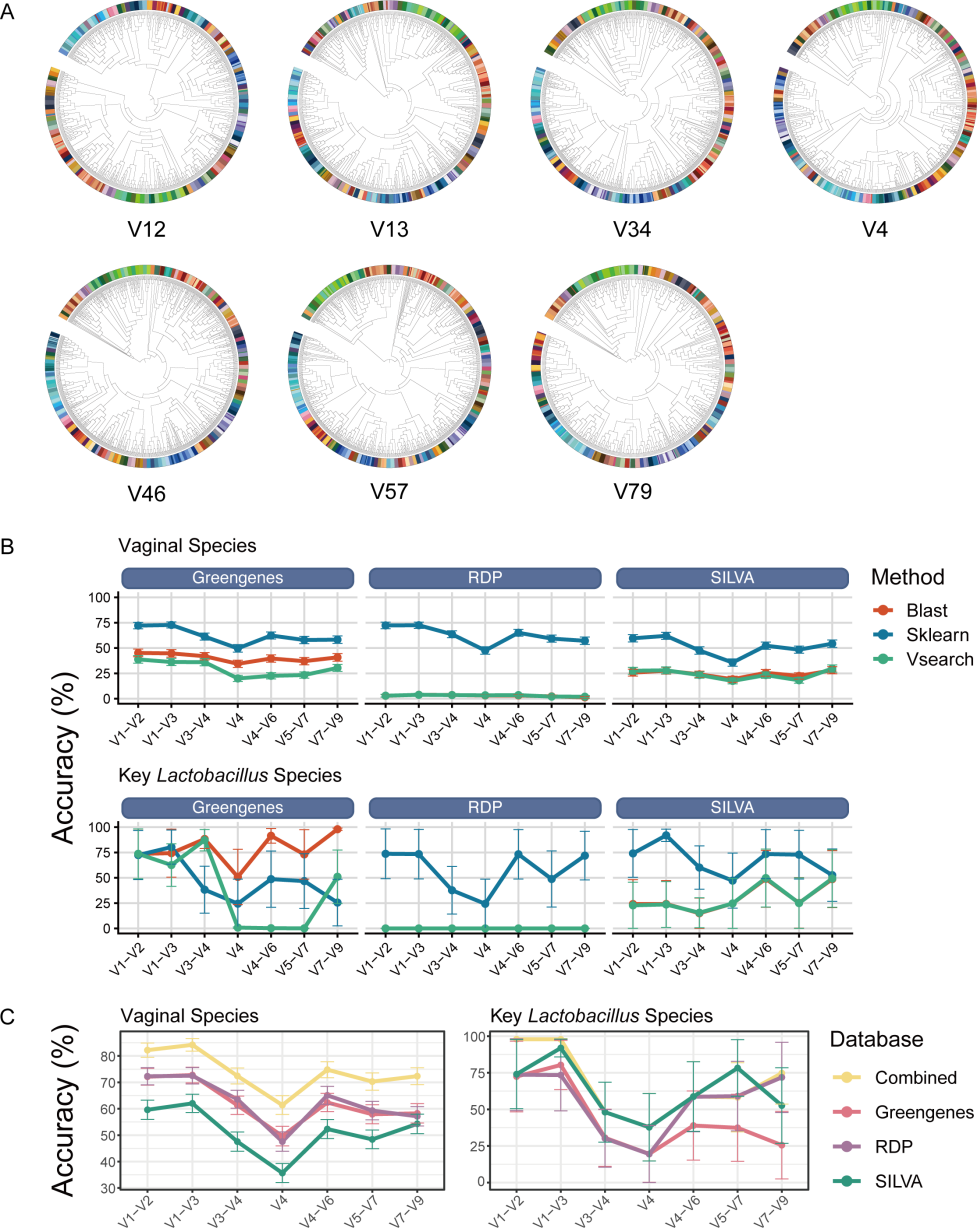
(**A**) Unrooted phylogenic tree of the amplicons generated in computational evaluation based on the sequences of vaginal species downloaded from NCBI with the primer sets targeting different 16S regions. The color of the node labels represents different bacterial species. (**B**) Classification accuracy of the pipelines comparing different classification methods and reference databases based on amplicons generated in computational evaluation, with all the species and the key *Lactobacillus* species (*L. crispatus*, *L. gasseri*, *L. iners*, and *L. jensenii*) alone presented, respectively. (**C**) Classification accuracy when combining the complementary results from Greengenes2, RDP, and SILVA together versus independently, with all the species and the key *Lactobacillus* species alone presented, respectively.

Of those vaginal bacterial species, 5,868,803 records of nucleotide sequences were downloaded from NCBI, resulting in 3,404,853 after deduplication (supplemental material). Uneven number of bacterial species were generated from the simulated amplification using Seqkit amplicon command with the primer sets (Table 1) targeting V1–V2, V1–V3, V3–V4, V4, V4–V6, V5–V7, and V7–V9 regions (Fig. S1). The potential heterogeneity of each 16S region can be indicated by the phylogeny (Fig. 1A). Significant variation in accuracy was presented by the pipelines that comparing different taxonomic classification algorithms (BLAST+, VSEARCH, and Sklearn) and reference databases (Greengenes2, SILVA, and RDP) when classifying the known bacterial amplicons of each 16S region. Pipeline V1–V3_Sklearn_Greengenes2 (72.77% ± 2.98%) slightly outperformed the Pipeline V1–V3_Sklearn_RDP (72.50% ± 3.09%) and achieved the highest classification accuracy. However, Pipeline V7–V9_BLAST_Greengenes2 (97.93% ± 1.22%) performed much better than the rest pipelines in distinguishing the key *Lactobacillus* species (*L. crispatus*, *L. gasseri*, *L. iners*, and *L. jensenii*), which is of vital importance in classifying vaginal microbiota (Fig. 1B). Each database has its own distinct advantages for classifying specific vaginal species when employing Sklearn (Fig. S2). Therefore, combining the classification results of the databases, by simply applying the most accurate one for specific species at specific 16S region, significantly improved the accuracy for the classification of all the species (82.19% ± 2.69% for V1–V2 and 84.20% ± 2.39% for V1–V3) and *Lactobacillus* species alone (97.90% ± 0.36% for V1–V2 and 97.83% ± 0.54% for V1–V3) (Fig. 1C).

At the V1–V3 region, RDP has high resolution for the classification of *Anaerococcus obesiensis* (45), *Atopobium vaginae* (46), *Enterococcus faecium* (47), *Fusobacterium nucleatum* (48), *Gardnerella vaginalis* (49), *L. gasseri* (50), *Gemella asaccharolytica* (7), *Murdochiella asaccharolytica* (51), *Mycoplasma hominis* (52), *Streptococcus mitis* (53), and *Ureaplasma urealyticum* (12). SILVA compensates in identifying BVAB1 (6), *L. jensenii* (54), *Mobiluncus curtisii* (55), *Sneathia amnii* (56), and *U. parvum* (12). Moreover, Greengenes2 increased the classification accuracy of *Bifidobacterium breve* (57), *Mycoplasma girerdii* (58) as well as novel vaginal species *Megasphaera lornae*, *Megasphaera hutchinsoni*, and *Megasphaera vaginalis* (59). These bacterial species were proven to have unignorable impact on human vaginal health.

Given that the redundancy of NCBI nucleotide database and that there were only 144 sharing species were amplified jointly by all seven primer sets (Fig. S1), we next employed experimental amplification and sequencing to validate the results from the computational evaluation.

### Full-length 16S rRNA gene sequencing of the vaginal mock samples

Based on the results of microecological assessment, HPV genotypes and other sexually transmitted pathogens detections, 115 participants with their vaginal swabs were selected including healthy women and those with vaginal dysbiosis, HPV, and other sexually transmitted infections (supplemental material). All vaginal swabs were mixed and evenly divided to create mock communities, of which 10 samples were randomly selected and sent for full-length 16S rRNA gene sequencing.

The representative sequences, which length ranged from 1,392 to 1,497 bp (Fig. S3A), were aligned and manually annotated against the NCBI Nucleotide Sequence Database. Out of the 162 vaginal bacterial species, 47 were detected in the mock samples, accounting for an average relative abundance of 94.24% ± 00.41% (Fig. 3A and B).

Based on the full-length 16S rRNA gene representative sequences, we similarly performed computational amplifications targeting the same 16S regions as above (Table 1). All sequences were successfully amplified by seven primer sets, respectively. The heterogeneity in length of the amplicons was comparable to those generated from NCBI downloaded sequences (Fig. S3B).

**Fig 2:**
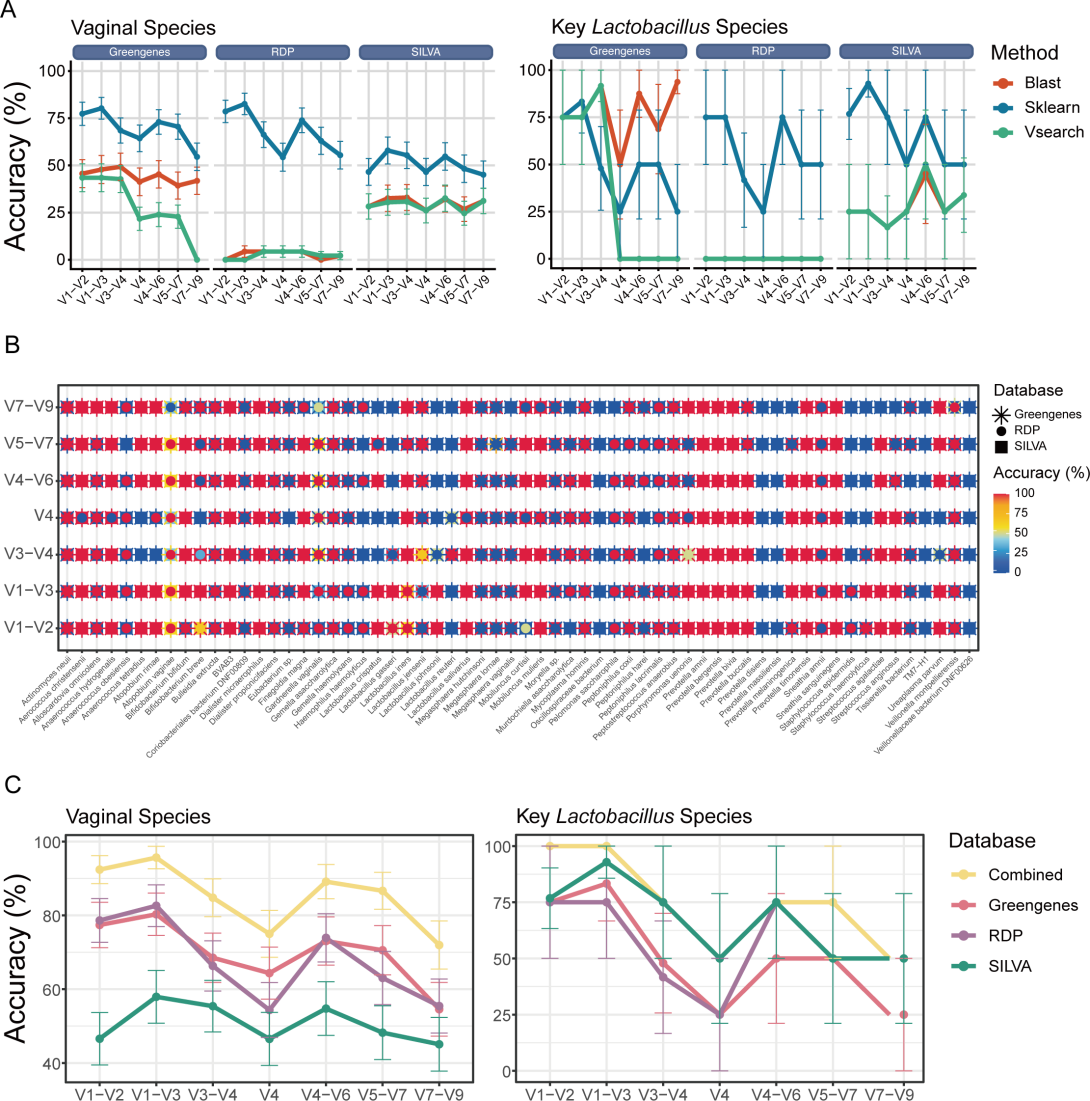
(**A**) Classification accuracy of the pipelines comparing different classification methods and reference databases based on the amplicons generated computationally from the 16S full-length sequencing data of the mock samples, with the vaginal species and the key *Lactobacillus* species presented, respectively. (**B**) Classification accuracy of each database on the amplicons targeting different 16S regions. All species that present in the representative sequences of 16S full-length sequencing data were shown. The asterisk, the circle, and the square represent the classification accuracy applying Greengenes2, RDP, and SILVA, respectively. (**C**) Classification accuracy on the amplicons generated computationally from the 16S full-length sequencing data when combining the complementary classification results from Greengenes2, RDP, and SILVA together versus independently, with all the species and the key *Lactobacillus* species alone presented, respectively.

Similarly, combinations of different reference databases and taxonomic classification methods were also applied. Similarly, Pipeline V1–V3_Sklearn_RDP had the highest accuracy for classifying all the detected species (74.19% ± 5.60%) (Fig. S4), the vaginal species (82.61% ± 5.65%). While the Pipeline V7–V9_BLAST_Greengenes had the highest accuracy for classifying key *Lactobacillus* species (93.75% ± 6.25%), slightly exceeded the Pipeline V1–V3_Sklearn_SILVA (92.86% ± 7.14%) (Fig. 2A). Expectably, after combining the complementary annotations from RDP and SILVA for specific species (Fig. 2B), the accuracy considerably increased to 90.32% ± 3.79% for all the species, 95.65% ± 3.04% for the vaginal bacterial species and 100% for the key *Lactobacillus* species (Pipeline V1–V3_Sklearn_Combined) (Fig. 2C). Overall, Pipeline V1–V3_Sklearn_Combined was further proved to be optimal for the taxonomic classification of vaginal microbiota data.

### Partial 16S rRNA gene sequencing of the vaginal mock samples

**Fig 3:**
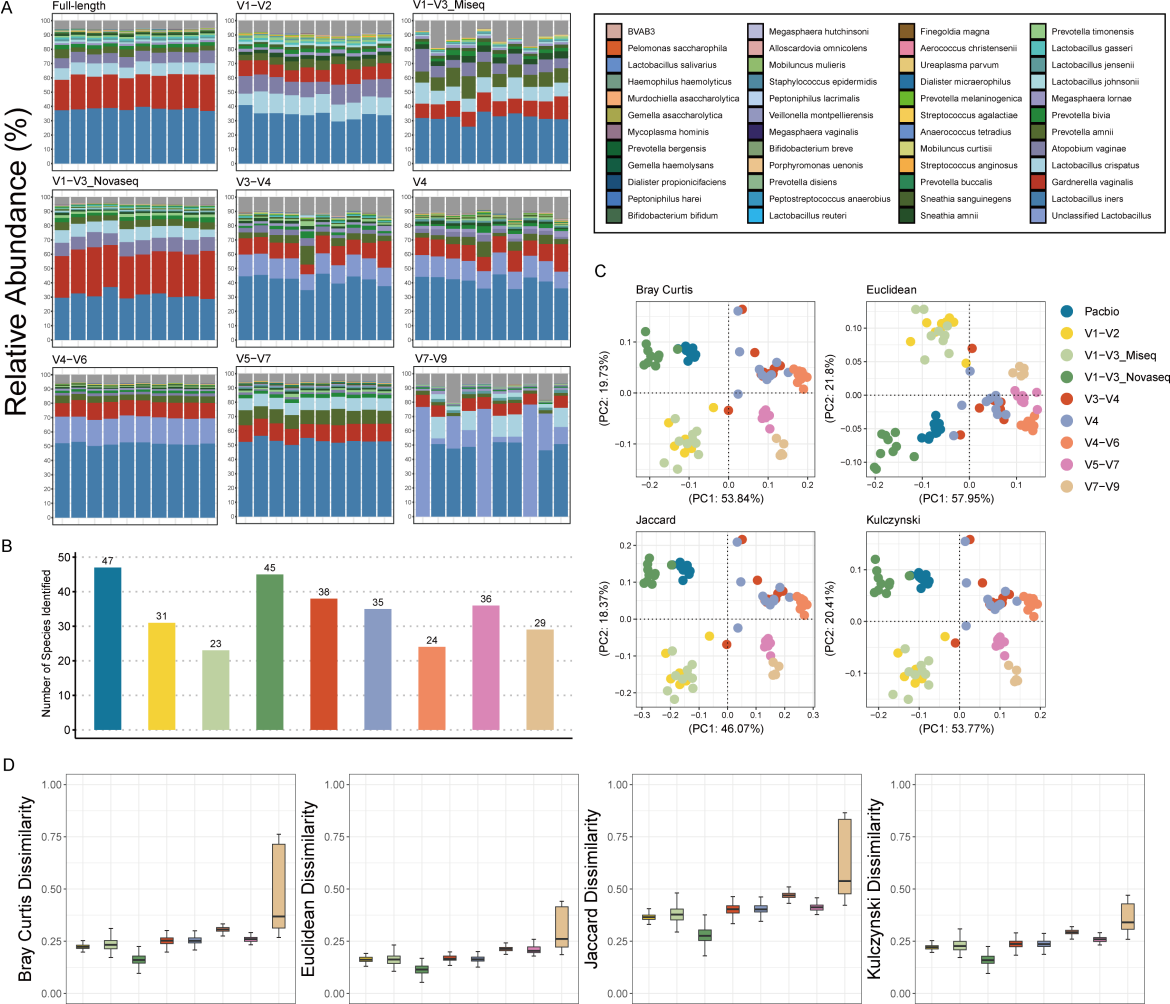
(**A**) Taxa stacked bar plots of the mock samples amplified experimentally with primer sets targeting different 16S regions, in which the vaginal bacterial species identified by the full-length sequencing are highlighted with different colors whereas other species were colored with gray. (**B**) Numbers of vaginal species identified by sequencing of each 16S region. For the partial 16S sequencing data, only the vaginal species which identified by the 16S full-length data could be included. (**C**) Principal coordinates analysis (PCoA) applying different distance algorithms, based on microbial profile of the mock samples amplified experimentally with primer sets targeting different 16S regions. (**D**) Dissimilarity applying different distance algorithms, based on species-level microbial profile of the mock samples amplified experimentally with primer sets targeting different partial 16S regions compared with the ones sequenced with 16S full-length.

To further solidate the conclusion drawn from the computational evaluation and 16S full-length sequencing. Another 80 mock samples were randomly picked and experimentally amplified using the primer sets in Table 1 for partial 16S rRNA gene sequencing. Unexpectedly, the V1–V3 seemed not an ideal region as we expected for its distorted microbial profile and the number of vaginal species identified, compared to that from the amplicons of the 16S full-length sequencing data. The situation improved when Illumina Novaseq PE250 was employed to sequence the amplicons of the mock samples generated from the V1–V3 primer set, while only the forward reads (223 bp) were applied without merging due to the insufficient sequencing length (Fig. 3A and B).

To determine which primer set has the most similar microbial profile to the full-length sequencing, the distance between each sample was calculated and PCoA analysis was performed based on the species-level relative abundance matrix, which demonstrated the smallest dissimilarity between the data of the V1–V3 forward reads and the full-length sequencing data (Fig. 3C and D). To verify this finding, we truncate the V1–V3 amplicons of the NCBI data, the 16S full-length data and the Illumina Miseq forward reads to 223 bp, simulating the forward reads from Illumina Novaseq. The classification performance and profile remained considerable after the truncation on the V1–V3 amplicons (Fig. S5 and 6).

Overall, through experimentally amplification and sequencing of the mock samples targeting different 16S regions, we finally confirmed our pipeline. Sequencing with Illumina Novaseq PE250 and applying only the forward reads seems a better choice than the pair-end reads merging strategy with Illumina Miseq PE300.

### Application and validation in a multicenter cohort

**Fig 4:**
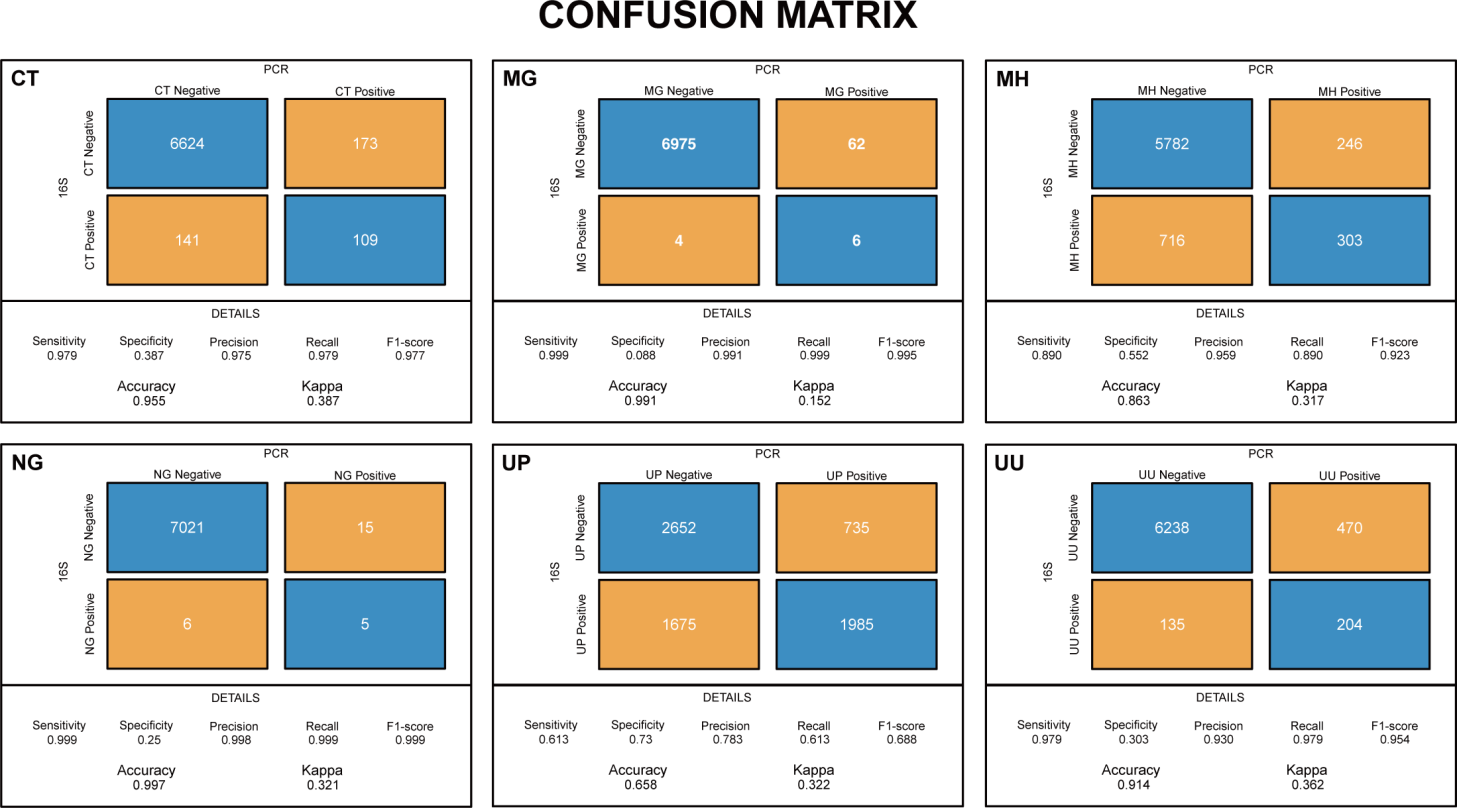
Confusion matrix of the STI pathogens with the PCR result as the reference test and 16S rRNA gene sequencing result (relative abundance more than or equal to 0.1% as positive, less than 0.1% as negative) as the predicted test. The sensitivity, specificity, precision, recall, F1-score, accuracy, and kappa were calculated. STI pathogens abbreviations: CT, *Chlamydia trachomatis*; MH, *Mycoplasma hominis*; MG, *Mycoplasmoides genitalium*; NG, *Neisseria gonorrhoeae*; UP, *Ureaplasma parvum*; UU, *Ureaplasma urealyticum*.

We further applied the pipeline in the analysis of the 16S sequencing data from our multicenter cohort with 7,076 women in reproductive age across China. To validate the species-level classification of the 16S sequencing data, we extracted the PCR results of the STI pathogens, including *Chlamydia trachomatis*, *Mycoplasma hominis*, *Mycoplasmoides genitalium*, *Neisseria gonorrhoeae*, *U. parvum*, and *U. urealyticum* (supplemental material). Confusion matrix and related metrics were applied, and the results showed that the performance of our pipeline to detect the STI pathogens except for *U. parvum* were excellent in F1-score, sensitivity, precision, recall, accuracy, but deficient in specificity and kappa (Fig. 4).

The PCR results for *U. parvum* may underestimated its presence, as it only detected the four known serotypes (*U. parvum* 1, *U. parvum* 3, *U. parvum* 6, and *U. parvum* 14) separately. This could explain the unsatisfactory performance of our pipeline to match the PCR result for this species.

Overall, the application of our pipeline in the cohort further proved its robustness in the species-level classification of some STI pathogens in vaginal microbiota.

## DISCUSSION

Accumulating studies have proven specific vaginal bacterial species to play vital roles in human vaginal niche. It is crucially important to comprehensively evaluate the methodology and achieve high species-level resolution for the analysis of vaginal microbiota NGS data. In this study, through computational and experimental evaluations, we have determined the optimal pipeline to have considerable performance in the species-level classification for the vaginal microbiota. The optimal pipeline consists of the 16S V1–V3 region to be amplified and sequenced (sequencing with Illumina Novaseq and applied only the forward reads), Sklearn as the taxonomic classification method and the complementary combination of Greengenes2, SILVA, and RDP as the reference database.

Targeting all the hypervariable regions in 16S rRNA gene, seven primer sets were applied in the computational and experimental analysis, of which all were used in previous studies on microbiota. Primer sets targeting the V1–V3 (24), V3–V4 (25), and V4 (23) regions were widely used in studies on vaginal microbiota and were proven to have high resolution in species-level analysis, and the V1–V3 was further validated in this study to be optimal. However, sequencing targeting the V3–V4 region underestimated the profile of *G. vaginalis* (Fig. 3A), which was a common problem among all the primer sets except for the V1–V3 and was contrary to Graspeuntner's findings (25). However, this controversy is a validation for the mixed primer 27F (31, 32) to facilitate the broad amplification of different vaginal species. The other limitation for sequencing the V3–V4 region was the inability to identify *L. crispatus*, and it was a flaw for the V4 region either, which was in conflict with Van Der Pol's findings (23) but was proposed in O'Callaghan's study (21) that the cost of sequencing the V4 region is the reduced sensitivity in diﬀerentiating *Lactobacillus* species.

Greengenes2, SILVA and RDP, which are commonly used in microbiota studies, were compared in this study. Our results indicated that Greengenes2 is suitable for classifying some specific vaginal bacterial species, which is a significant improvement to its previous version, which is not suitable for identifying vaginal species, according to Van Der Pol's report (23). Greengenes2 is recently reported to have much larger phylogenetic coverage than other resources (60), while SILVA has the highest sequence quality, RDP has high 16S sequence coverage (61). Each database has its own advantages in classifying specific vaginal species, and their combination complements each other.

The V1–V3 data from Illumina Novaseq exceeded the other regions data from Illumina Miseq by detecting the most vaginal species and resembling the microbial profile of the full-length sequencing data the most, with barely utilizing the forward sequences. This brings about the controversy about the sequencing platform, that Illumina Miseq excels for its long sequencing length but was criticized for relatively low Q30 in the 3′ end of both the forward and the reverse reads. This results in fewer sequences passing the quality filtering or a low merging ratio due to truncation. In contrast, Illumina Novaseq produces higher-quality data and has much higher throughput and sequencing depth, which may possibly contribute to the outperformance of the forward reads of the V1–V3 region.

We acknowledge several limitations to this study. First, we selected vaginal bacterial species for analysis based on previous publications, which means our pipeline may possibly be unable to recognize other species as well as those rare species. Moreover, our pipeline failed to identify some of the vaginal species, which may not be very common in the vaginal niche according to current studies. For those were not successfully computationally amplified, the possible reason lies in that the sequences downloaded from NCBI may lacked the V1–V3 region. Finally, the gradually decreasing of the cost for 16S full-length or even metagenomic shotgun sequencing will eventually narrow the application of our pipeline.

### Conclusions

In the present study, we determined the optimal 16S region and taxonomic classification method and reference database for the 16S rRNA gene sequencing of vaginal microbiota, both computationally and experimentally. For the computational evaluation, we performed simulated amplification with seven primer sets targeting different 16S regions, based on the sequences of 162 vaginal bacterial species downloaded from NCBI, determining that the Pipeline V1–V3_Sklearn_Combined to have the highest accuracy for classifying the vaginal species. For the experimental analysis, we prepared the vaginal mock samples, on which 16S full-length sequencing was performed. By both computationally and experimentally amplifying the mock samples targeting different 16S regions, we further confirmed that V1–V3, Sklearn and Greengenes2, RDP combined with SILVA to be the optimal 16S sequencing region, classification method and reference database combination, respectively, and together as an optimal pipeline for the species-level classification of vaginal microbiota. Interestingly, sequencing with Illumina Novaseq PE250 targeting the V1–V3 region and applying only the forward reads seems a better choice than the pair-end reads merging strategy using Illumina Miseq PE300. Our study determined a pipeline that achieved high species-level resolution on vaginal microbiota even with short amplicons, which could be a promising approach to facilitate future studies.

## SUPPLEMENTAL MATERIAL

Fig. S1 msystems.01039-23-s0001.docx Number of species generated by different primer sets.

Fig. S2 msystems.01039-23-s0002.docx Classification accuracy employing Sklearn with Greengenes2, RDP, and SILVA on amplicons targeting different 16S regions of the vaginal bacterial species.

Fig. S3 msystems.01039-23-s0003.docx Sequence length of the representative sequences generated from the 16S full-length sequencing data of the mock samples and of the amplicons generated computationally from the 16S full-length sequencing data.

Fig. S4 msystems.01039-23-s0004.docx Classification accuracy.

Fig. S5 msystems.01039-23-s0005.docx Numbers of vaginal species, PCoA, and dissimilarity.

Fig. S6 msystems.01039-23-s0006.docx Classification accuracy of our pipeline on the V1-V3 forward amplicons (truncated the first 223 bases to simulate the forward pair-end sequencing data from Illumina Novaseq) generated computationally from the NCBI downloaded data.

Metadata msystems.01039-23-s0007.xlsx Metadata of the participants.

Table S1 msystems.01039-23-s0008.docx Vaginal bacterial species and source publications.

ASM does not own the copyrights to Supplemental Material that may be linked to, or accessed through, an article. The authors have granted ASM a non-exclusive, world-wide license to publish the Supplemental Material files. Please contact the corresponding author directly for reuse.
