# Supplementary material for: Species-level resolution for the vaginal microbiota with short amplicons
Source: mSystems. 2024 Jan 26;9(2):e01039-23. doi: 10.1128/msystems.01039-23 (PMC10878104; doi:10.1128/msystems.01039-23)
Supplement: Fig. S1 — Number of species generated by different primer sets. [file msystems.01039-23-s0001.docx]

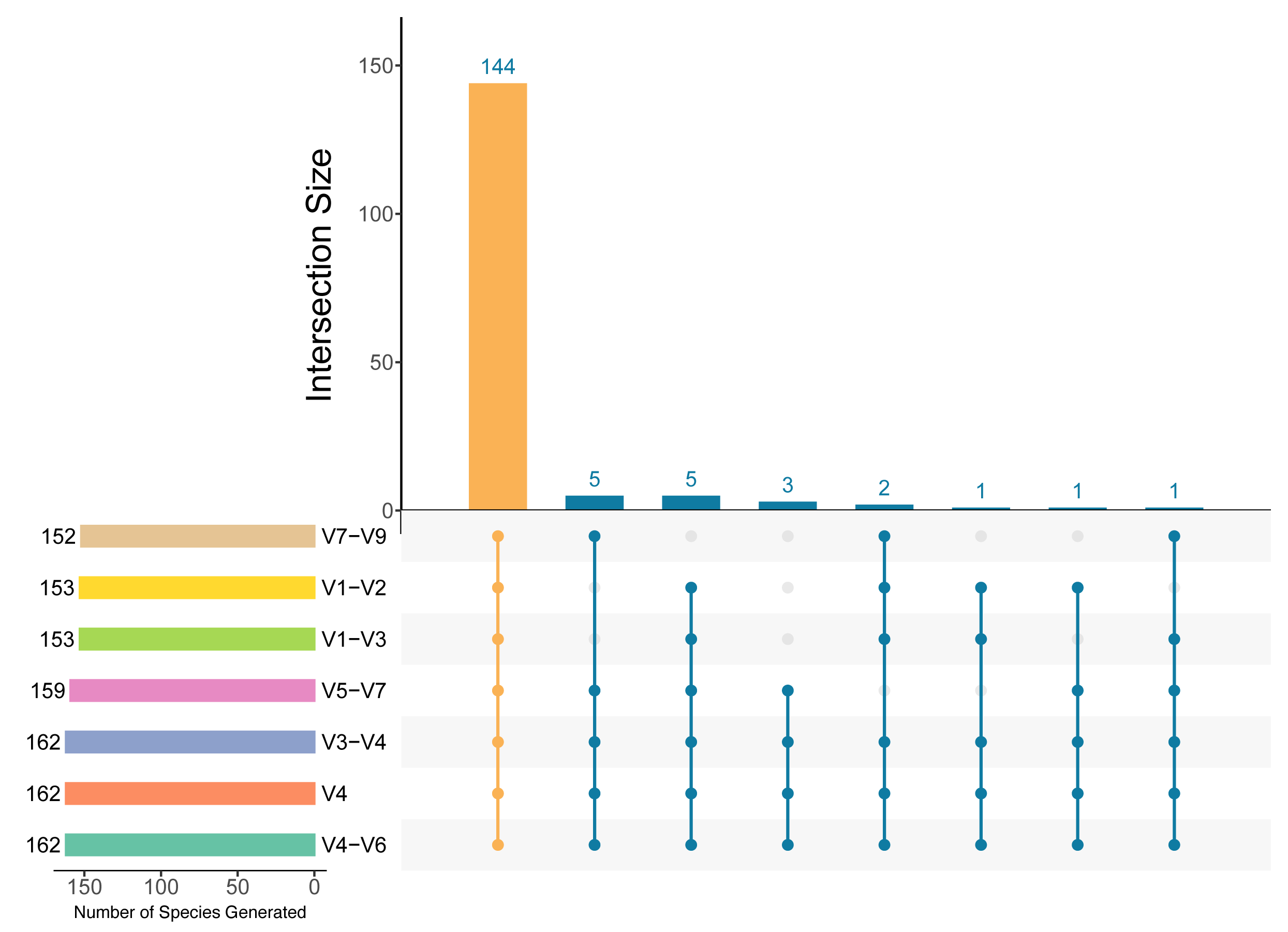


**Supplementary Figure 1.** The bar plots on the left show the number of species generated with the primer sets targeting different 16S regions. The plots on the right show the number of sharing species generated by different primer sets. The bar representing the 144 species which were jointly amplified by all seven primer sets were highlighted with orange.
