# Supplementary material for: Species-level resolution for the vaginal microbiota with short amplicons
Source: mSystems. 2024 Jan 26;9(2):e01039-23. doi: 10.1128/msystems.01039-23 (PMC10878104; doi:10.1128/msystems.01039-23)
Supplement: Fig. S2 — Classification accuracy employing Sklearn with Greengenes2, RDP, and SILVA on amplicons targeting different 16S regions of the vaginal bacterial species. [file msystems.01039-23-s0002.docx]

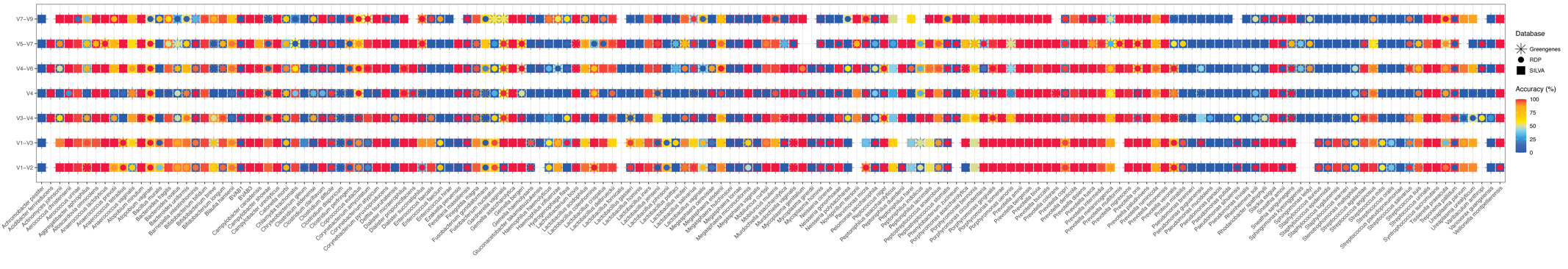


**Supplementary Figure 2.** Classification accuracy employing Sklearn with Greengenes2, RDP and SILVA on amplicons targeting different 16S regions of the vaginal bacterial species. The asterisk, the circle and the square represent the classification accuracy of Greengenes2, RDP and SILVA respectively.
