# Supplementary material for: Species-level resolution for the vaginal microbiota with short amplicons
Source: mSystems. 2024 Jan 26;9(2):e01039-23. doi: 10.1128/msystems.01039-23 (PMC10878104; doi:10.1128/msystems.01039-23)
Supplement: Fig. S3 — Sequence length of the representative sequences generated from the 16S full-length sequencing data of the mock samples and of the amplicons generated computationally from the 16S full-length sequencing data. [file msystems.01039-23-s0003.docx]

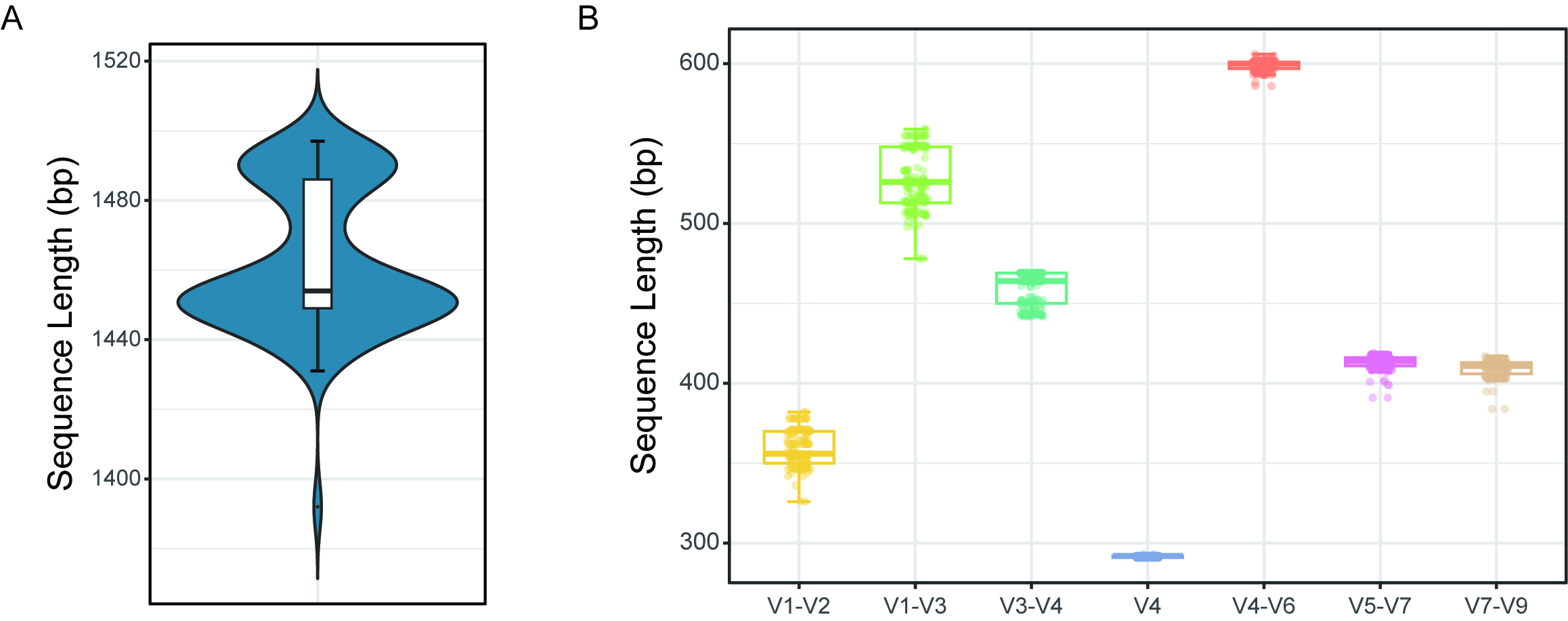


**Supplementary Figure 3.** (A) Sequence length of the representative sequences generated from the 16S full-length sequencing data of the mock samples. (B) Sequence length of the amplicons generated computationally from the 16S full-length sequencing data.
