# Supplementary material for: Species-level resolution for the vaginal microbiota with short amplicons
Source: mSystems. 2024 Jan 26;9(2):e01039-23. doi: 10.1128/msystems.01039-23 (PMC10878104; doi:10.1128/msystems.01039-23)
Supplement: Fig. S4 — Classification accuracy. [file msystems.01039-23-s0004.docx]

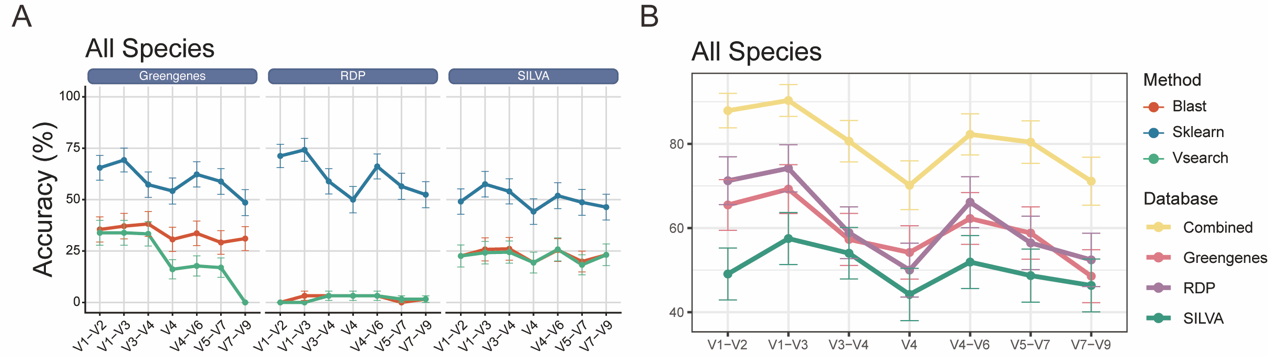


**Supplementary Figure 4.** (A) Classification accuracy of the pipelines comparing different classification methods and reference databases based on amplicons of all the detected species generated computationally from the 16S full-length sequencing data. (B) Classification accuracy when combining the complementary classification results from Greengenes2, RDP and SILVA together versus independently.
