# Supplementary material for: Species-level resolution for the vaginal microbiota with short amplicons
Source: mSystems. 2024 Jan 26;9(2):e01039-23. doi: 10.1128/msystems.01039-23 (PMC10878104; doi:10.1128/msystems.01039-23)
Supplement: Fig. S5 — Numbers of vaginal species, PCoA, and dissimilarity. [file msystems.01039-23-s0005.docx]

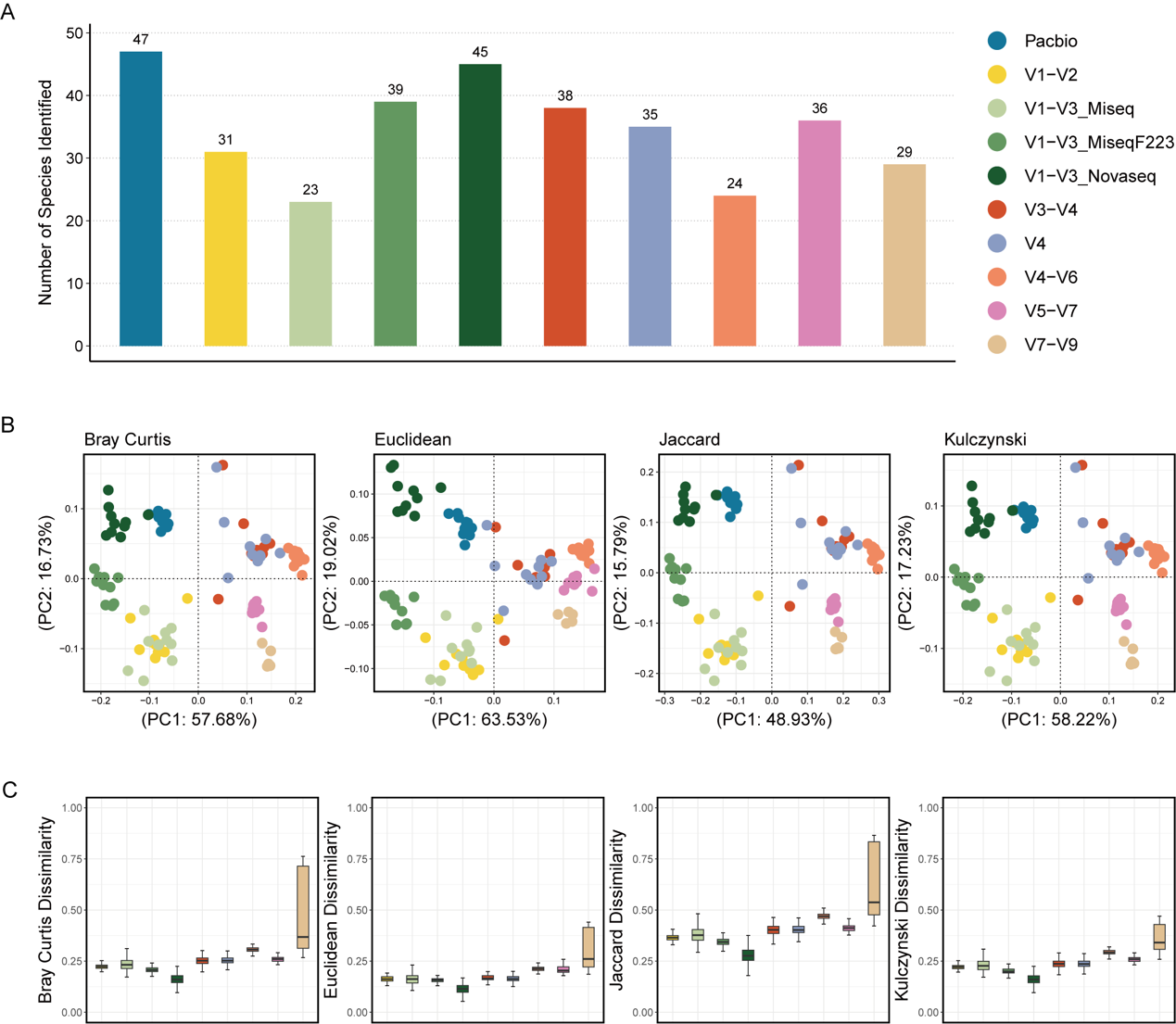


**Supplementary Figure 5.** (A) Numbers of vaginal species identified by sequencing of each 16S region. For the partial 16S sequencing data, only the vaginal species which identified by the 16S full-length data could be included. (B) Principal Coordinates Analysis (PCoA) applying different distance algorithms, based on microbial profile of the mock samples amplified experimentally with primer sets targeting different 16S regions. (C) Dissimilarity applying different distance algorithms, based on species-level microbial profile of the mock samples amplified experimentally with primer sets targeting different partial 16S regions compared with the ones sequenced with 16S full-length.
