# Supplementary material for: Species-level resolution for the vaginal microbiota with short amplicons
Source: mSystems. 2024 Jan 26;9(2):e01039-23. doi: 10.1128/msystems.01039-23 (PMC10878104; doi:10.1128/msystems.01039-23)
Supplement: Fig. S6 — Classification accuracy of our pipeline on the V1-V3 forward amplicons (truncated the first 223 bases to simulate the forward pair-end sequencing data from Illumina Novaseq) generated computationally from the NCBI downloaded data. [file msystems.01039-23-s0006.docx]

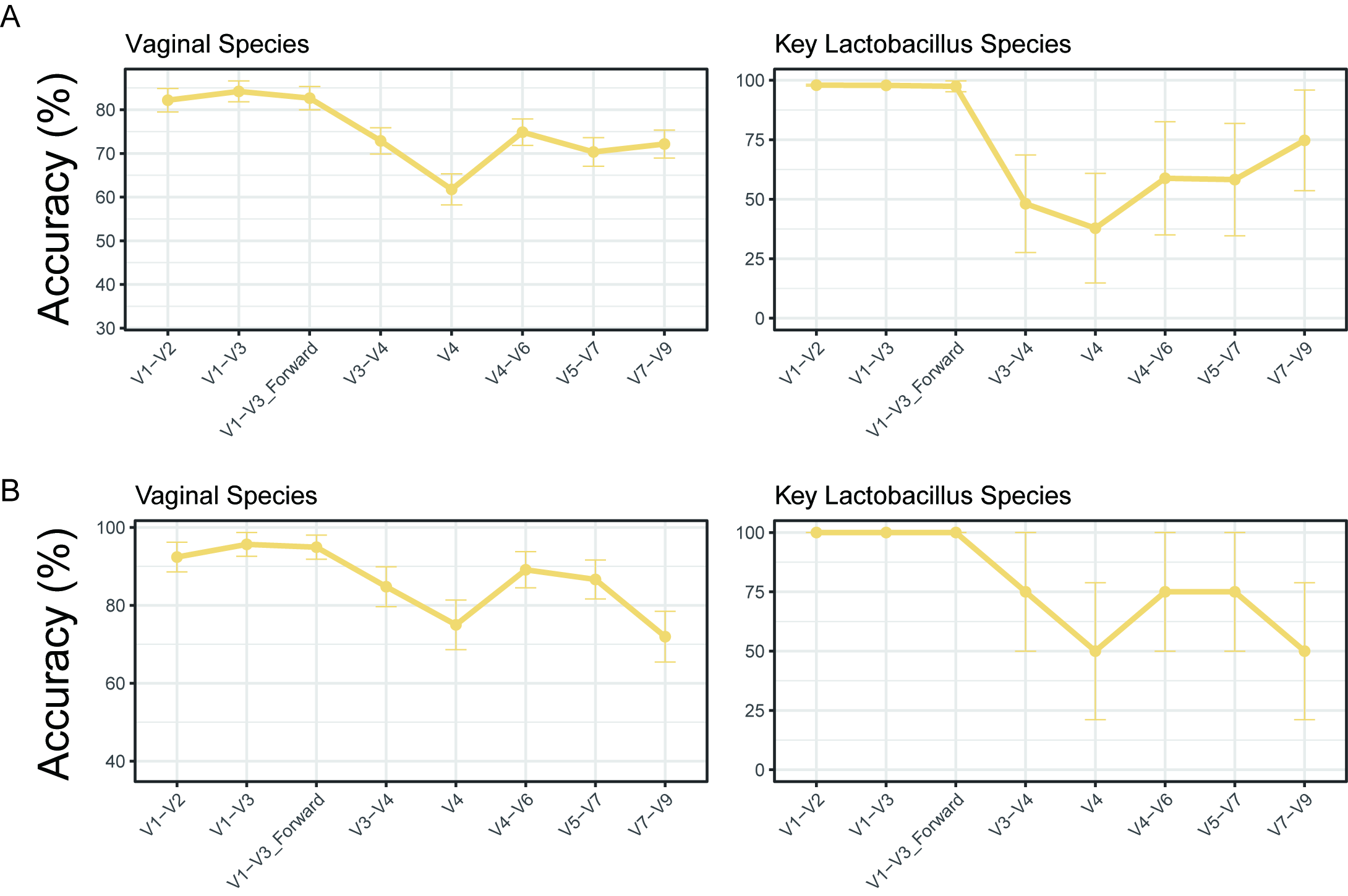


**Supplementary Figure 6.** Classification accuracy of our pipeline on the V1-V3 forward amplicons (truncated the first 223 bases to simulate the forward pair-end sequencing data from Illumina Novaseq) generated computationally from the NCBI downloaded data (A) and the 16S full-length sequencing data (B), compared with that of other partial 16S region, with all the vaginal species and the key *Lactobacillus* species alone presented respectively.
