# Supplementary material for: Species-level resolution for the vaginal microbiota with short amplicons
Source: mSystems. 2024 Jan 26;9(2):e01039-23. doi: 10.1128/msystems.01039-23 (PMC10878104; doi:10.1128/msystems.01039-23)
Supplement: Table S1 — Vaginal bacterial species and source publications. [file msystems.01039-23-s0008.docx]

| **Supplementary Table1.** Vaginal Bacterial Species and Source Publications | |  |
| --- | --- | --- |
| Bacterial Species | Publications | |
| *Achromobacter pestifer*^1^ | Filardo et al. 2019 | |
| *Acidibacter ferrireducens*^1^ | Filardo et al. 2019 | |
| *Actinomyces johnsonii*^2^ | Usyk et al. 2020 | |
| *Aerococcus christensenii*^3-6^ | Chao et al. 2021  McClelland et al. 2018  Romero et al. 2014  Mitra et al. 2015 | |
| *Aerococcus urinae*^7^ | Alioua et al. 2016 | |
| *Aggregatibacter aphrophilus*^1^ | Filardo et al. 2019 | |
| *Alloscardovia omnicolens*^3,8^ | Chao et al. 2021  Tabatabaei et al. 2019 | |
| *Anaerococcus lactolyticus*^6,9^ | Shrestha et al. 2018  Mitra et al. 2015 | |
| *Anaerococcus prevotii*^2,10^ | Usyk et al. 2020  Si et al. 2017 | |
| *Anaerococcus tetradius*^6,11,12^ | Dareng et al. 2016  Muzny et al. 2018  Mitra et al. 2015 | |
| *Anaerococcus vaginalis*^6,12^ | Muzny et al. 2018  Mitra et al. 2015 | |
| *Atopobium minutum*^2^ | Usyk et al. 2020 | |
| *Atopobium vaginae*^3,4,6,8,13,14^ | Mitra et al. 2015  McClelland et al. 2018  Dols et al. 2011  Onderdonk et al. 2016  Chao et al. 2021  Tabatabaei et al. 2019 | |
| *Bacillus muralis*^10^ | Si et al. 2017 | |
| *Bacteroides fragilis*^15^ | Borgdorff et al. 2014 | |
| *Bacteroides ovatus*^10^ | Si et al. 2017 | |
| *Bacteroides uniformis*^12^ | Muzny et al. 2018 | |
| *Barnesiella intestinihominis*^1^ | Filardo et al. 2019 | |
| *Biﬁdobacterium bifidum*^1,2^ | Usyk et al. 2020  Filardo et al. 2019 | |
| *Biﬁdobacterium breve*^6,8,16^ | Mitra et al. 2015  Huang et al. 2018  Tabatabaei et al. 2019 | |
| *Bifidobacterium longum*^2,8^ | Usyk et al. 2020  Tabatabaei et al. 2019 | |
| *Blautia hansenii*^2^ | Usyk et al. 2020 | |
| *BVAB1*^14,17^ | Onderdonk et al. 2016  Fettweis et al. 2019 | |
| *BVAB3*^1,14^ | Filardo et al. 2019  Onderdonk et al. 2016 | |
| *Campylobacter canadensis*^2^ | Usyk et al. 2020 | |
| *Campylobacter showae*^1^ | Filardo et al. 2019 | |
| *Campylobacter ureolyticus*^2^ | Usyk et al. 2020 | |
| *Catonella morbi*^2,6^ | Mitra et al. 2015  Usyk et al. 2020 | |
| *Chlamydia trachomatis*^18^ | Anahtar et al. 2018 | |
| *Chryseobacterium gleum*^2^ | Usyk et al. 2020 | |
| *Clostridium aldenense*^6^ | Mitra et al. | |
| *Clostridium clariflavum*^2^ | Usyk et al. 2020 | |
| *Clostridium difficile*^2^ | Usyk et al. 2020 | |
| *Clostridium disporicum*^3^ | Chao et al. 2021 | |
| *Clostridium perfringens*^1^ | Filardo et al. 2019 | |
| *Coprococcus eutactus*^2^ | Usyk et al. 2020 | |
| *Corynebacterium amycolatum*^4^ | McClelland et al. 2018 | |
| *Corynebacterium atypicum*^2^ | Usyk et al. 2020 | |
| *Corynebacterium pyruviciproducens*^6^ | Mitra et al. 2015 | |
| *Delftia tsuruhatensis*^1^ | Filardo et al. 2019 | |
| *Dialister micraerophilus^4,6,10,15,17^* | Mitra et al. 2015  McClelland et al. 2018  Borgdorff et al. 2014  Si et al. 2017  Fettweis et al. 2019 | |
| *Dialister propionicifaciens*^12,15^ | Muzny et al. 2018  Borgdorff et al. 2014 | |
| *Dialister succinatiphilus*^2^ | Usyk et al. 2020 | |
| *Enterococcus faecalis*^7,19^ | Alioua et al. 2016  Oh et al. 2015 | |
| *Enterococcus faecium*^7^ | Alioua et al. 2016 | |
| *Enterococcus hirae*^7^ | Alioua et al. 2016 | |
| *Ezakiella peruensis*^1^ | Filardo et al. 2019 | |
| *Fenollaria massiliensis*^1^ | Filardo et al. 2019 | |
| *Finegoldia magna*^5,6,8,20^ | Mitra et al. 2015  Godoy-Vitorino et al. 2018  Romero et al. 2014  Tabatabaei et al. 2019 | |
| *Fusobacterium gonidiaformans*^21^ | Anahtar et al. 2015 | |
| *Fusobacterium nucleatum*^6,22^ | Mitra et al. 2015  Ling et al. 2010 | |
| *Gardnerella vaginalis*^6,8,13,14^ | Mitra et al. 2015  Dols et al. 2011  Onderdonk et al. 2016  Tabatabaei et al. 2019 | |
| *Gemella asaccharolytica*^1,4,16^ | Huang et al. 2018  McClelland et al. 2018  Filardo et al. 2019 | |
| *Gemella bergeri*^2^ | Usyk et al. 2020 | |
| *Gemella haemolysans*^6^ | Mitra et al. 2015 | |
| *Gluconacetobacter takamatsuzukensis*^1^ | Filardo et al. 2019 | |
| *Haemophilus haemolyticus*^19^ | Oh et al. 2015 | |
| *Haemophilus influenzae*^23^ | Cox et al. 2002 | |
| *Hydrogenophaga flava*^1^ | Filardo et al. 2019 | |
| *Lachnobacterium bovis*^1,21^ | Anahtar et al. 2015  Filardo et al. 2019 | |
| *Lactobacillus acidophilus*^7,8^ | Alioua et al. 2016  Tabatabaei et al. 2019 | |
| *Lactobacillus coleohominis*^1,6,12^ | Mitra et al. 2015  Muzny et al. 2018  Filardo et al. 2019 | |
| *Lactobacillus crispatus*^8,18,24^ | Anahtar et al. 2018  Gajer et al. 2012  Tabatabaei et al. 2019 | |
| *Lactobacillus delbrueckii*^7,17^ | Alioua et al. 2016  Fettweis et al. 2019 | |
| *Lactobacillus fornicalis*^19^ | Oh et al. 2015 | |
| *Lactobacillus gasseri*^8,18,24^ | Anahtar et al. 2018  Gajer et al. 2012  Tabatabaei et al. 2019 | |
| *Lactobacillus hominis*^16^ | Huang et al. 2018 | |
| *Lactobacillus iners*^3,8,18,24^ | Anahtar et al. 2018  Gajer et al. 2012  Chao et al. 2021  Tabatabaei et al. 2019 | |
| *Lactobacillus jensenii*^3,8,18,24^ | Anahtar et al. 2018  Gajer et al. 2012  Chao et al. 2021  Tabatabaei et al. 2019 | |
| *Lactobacillus johnsonii*^8^ | Tabatabaei et al. 2019 | |
| *Lactobacillus psittaci*^19^ | Oh et al. 2015 | |
| *Lactobacillus reuteri*^3,4,16,21,24^ | Anahtar et al. 2015  Huang et al. 2018  McClelland et al. 2018  Gajer et al. 2012  Chao et al. 2021 | |
| *Lactobacillus salivarius*^15,16^ | Huang et al. 2018  Borgdorff et al. 2014 | |
| *Lactobacillus vaginalis*^4-6,15,19,24^ | Mitra et al. 2015  McClelland et al. 2018  Borgdorff et al. 2014  Gajer et al. 2012  Oh et al. 2015  Romero et al. 2014 | |
| *Megasphaera cerevisiae*^16^ | Huang et al. 2018 | |
| *Megasphaera elsdenii*^2^ | Usyk et al. 2020 | |
| *Megasphaera hutchinsoni*^25^ | Srinivasan et al. 2019 | |
| *Megasphaera lornae*^25^ | Srinivasan et al. 2019 | |
| *Megasphaera micronuciformis*^1^ | Filardo et al. 2019 | |
| *Megasphaera vaginalis*^25^ | Srinivasan et al. 2019 | |
| *Mobiluncus curtisii*^13,14^ | Dols et al. 2011  Onderdonk et al. 2016 | |
| *Mobiluncus mulieris*^13,14^ | Dols et al. 2011  Onderdonk et al. 2016 | |
| *Murdochiella asaccharolytica*^26^ | Diop et al. 2018 | |
| *Murdochiella vaginalis*^26^ | Diop et al. 2018 | |
| *Mycoplasma genitalium*^14,18^ | Anahtar et al. 2018  Onderdonk et al. 2016 | |
| *Mycoplasma girerdii*^17,27^ | Fettweis et al. 2019  Margarita et al. 2022 | |
| *Mycoplasma hominis*^4,6,14,19^ | Mitra et al. 2015  McClelland et al. 2018  Oh et al. 2015  Onderdonk et al. 2016 | |
| *Neisseria cinerea*^1^ | Filardo et al. 2019 | |
| *Neisseria gonorrhoeae*^2,14,18^ | Usyk et al. 2020  Anahtar et al. 2018  Onderdonk et al. 2016 | |
| *Neisseria polysaccharea*^1^ | Filardo et al. 2019 | |
| *Novispirillum itersonii*^1^ | Filardo et al. 2019 | |
| *Parvimonas micra^4-6,11,15^* | Mitra et al. 2015  McClelland et al. 2018  Borgdorff et al. 2014  Dareng et al. 2016  Romero et al. 2014 | |
| *Pelomonas saccharophila*^1^ | Filardo et al. 2019 | |
| *Peptococcus niger*^6^ | Mitra et al. 2015 | |
| *Peptoniphilus asaccharolyticus*^10,11^ | Dareng et al. 2016  Si et al. 2017 | |
| *Peptoniphilus duerdenii*^1^ | Filardo et al. 2019 | |
| *Peptoniphilus harei*^2,6,12^ | Mitra et al. 2015  Usyk et al. 2020  Muzny et al. 2018 | |
| *Peptoniphilus indolicus*^2^ | Usyk et al. 2020 | |
| *Peptoniphilus lacrimalis*^5,6^ | Mitra et al. 2015  Romero et al. 2014 | |
| *Peptostreptococcus anaerobius*^6,12,14,16^ | Mitra et al. 2015  Huang et al. 2018  Muzny et al. 2018  Onderdonk et al. 2016 | |
| *Peptostreptococcus stomatis*^6^ | Mitra et al. 2015 | |
| *Phenylobacterium zucineum*^1^ | Filardo et al. 2019 | |
| *Porphyromonas asaccharolytica*^15,28^ | Borgdorff et al. 2014  Lithgow et al. 2022 | |
| *Porphyromonas bennonis*^2,6,28^ | Mitra et al. 2015  Usyk et al. 2020  Lithgow et al. 2022 | |
| *Porphyromonas circumdentaria*^2^ | Usyk et al. 2020 | |
| *Porphyromonas gingivalis*^2,28^ | Usyk et al. 2020  Lithgow et al. 2022 | |
| *Porphyromonas somerae*^6,28^ | Mitra et al. 2015  Lithgow et al. 2022 | |
| *Porphyromonas uenonis*^15,28^ | Borgdorff et al. 2014  Lithgow et al. 2022 | |
| *Prevotella amnii*^2,3,6,15,17^ | Mitra et al. 2015  Usyk et al. 2020  Borgdorff et al. 2014  Chao et al. 2021  Fettweis et al. 2019 | |
| *Prevotella bergensis*^2,15^ | Usyk et al. 2020  Borgdorff et al. 2014 | |
| *Prevotella bivia*^3,6,8,13,14,18^ | Mitra et al. 2015  Anahtar et al. 2018  Dols et al. 2011  Onderdonk et al. 2016  Chao et al. 2021  Tabatabaei et al. 2019 | |
| *Prevotella buccalis*^6,15,17,19^ | Mitra et al. 2015  Borgdorff et al. 2014  Oh et al. 2015  Fettweis et al. 2019 | |
| *Prevotella colorans*^1^ | Filardo et al. 2019 | |
| *Prevotella copri*^10,21^ | Anahtar et al. 2015  Si et al. 2017 | |
| *Prevotella denticola*^1,19^ | Filardo et al. 2019  Oh et al. 2015 | |
| *Prevotella disiens*^6,13-15^ | Mitra et al. 2015  Borgdorff et al. 2014  Dols et al. 2011  Onderdonk et al. 2016 | |
| *Prevotella fusca*^16^ | Huang et al. 2018 | |
| *Prevotella intermedia*^17,21^ | Anahtar et al. 2015  Fettweis et al. 2019 | |
| *Prevotella melaninogenica*^3,5,6,18,21^ | Mitra et al. 2015  Anahtar et al. 2015  Anahtar et al. 2018  Romero et al. 2014  Chao et al. 2021 | |
| *Prevotella multiformis*^1,6^ | Mitra et al. 2015  Filardo et al. 2019 | |
| *Prevotella nigrescens*^13^ | Dols et al. 2011 | |
| *Prevotella oris*^1^ | Filardo et al. 2019 | |
| *Prevotella paludivivens*^2^ | Usyk et al. 2020 | |
| *Prevotella ruminicola*^15^ | Borgdorff et al. 2014 | |
| *Prevotella timonensis*^4,6,8,29^ | Mitra et al. 2015  McClelland et al. 2018  Mitra et al. 2020  Tabatabaei et al. 2019 | |
| *Prevotella veroralis*^2,6^ | Mitra et al. 2015  Usyk et al. 2020 | |
| *Proteus mirabilis*^30^ | Schaffer et al. 2015 | |
| *Pseudomonas brenneri*^16^ | Huang et al. 2018 | |
| *Pseudomonas guguanensis*^1^ | Filardo et al. 2019 | |
| *Pseudomonas mucidolens*^16^ | Huang et al. 2018 | |
| *Pseudomonas panacis*^6^ | Mitra et al. 2015 | |
| *Pseudomonas putida*^16^ | Huang et al. 2018 | |
| *Pseudomonas sihuiensis*^1^ | Filardo et al. 2019 | |
| *Pseudomonas trivialis*^19^ | Oh et al. 2015 | |
| *Rheinheimera soli*^6^ | Mitra et al. 2015 | |
| *Rhodanobacter spathiphylli*^6^ | Mitra et al. 2015 | |
| *Slackia exigua*^1^ | Filardo et al. 2019 | |
| *Sneathia amnii*^3,8,13,14,17^ | Dols et al. 2011  Onderdonk et al. 2016  Chao et al. 2021  Fettweis et al. 2019  Tabatabaei et al. 2019 | |
| *Sneathia sanguinegens*^3,6,13,14,18^ | Mitra et al. 2015  Anahtar et al. 2018  Dols et al. 2011  Onderdonk et al. 2016  Chao et al. 2021 | |
| *Sphingomonas kyeonggiensis*^1^ | Filardo et al. 2019 | |
| *Sphingomonas leidyi*^2^ | Usyk et al. 2020 | |
| *Staphylococcus aureus*^6,14^ | Mitra et al. 2015  Onderdonk et al. 2016 | |
| *Staphylococcus epidermidis*^19^ | Oh et al. 2015 | |
| *Staphylococcus lugdunensis*^6,12^ | Mitra et al. 2015  Muzny et al. 2018 | |
| *Staphylococcus warneri*^6^ | Mitra et al. 2015 | |
| *Stenotrophomonas maltophilia*^6^ | Mitra et al. 2015 | |
| *Streptococcus agalactiae*^6,8,31^ | Mitra et al. 2015  Vornhagen et al. 2018  Tabatabaei et al. 2019 | |
| *Streptococcus anginosus*^8,12,16^ | Huang et al. 2018  Muzny et al. 2018  Tabatabaei et al. 2019 | |
| *Streptococcus mitis*^6^ | Mitra et al. 2015 | |
| *Streptococcus oralis*^16^ | Huang et al. 2018 | |
| *Streptococcus pseudopneumoniae*^19^ | Oh et al. 2015 | |
| *Streptococcus salivarius*^6^ | Mitra et al. 2015 | |
| *Streptococcus suis*^16^ | Huang et al. 2018 | |
| *Streptococcus urinalis*^6^ | Mitra et al. 2015 | |
| *Syntrophococcus sucromutans*^1^ | Filardo et al. 2019 | |
| *Tissierella praeacuta*^21^ | Anahtar et al. 2015 | |
| *Treponema pallidum*^2^ | Usyk et al. 2020 | |
| *Ureaplasma parvum*^6,16^ | Mitra et al. 2015  Huang et al. 2018  Rittenschober-Böhm et al. 2019 | |
| *Ureaplasma urealyticum*^14^ | Onderdonk et al. 2016 | |
| *Varibaculum anthropi*^1^ | Filardo et al. 2019 | |
| *Variovorax guangxiensis*^1^ | Filardo et al. 2019 | |
| *Veillonella montpellierensis*^3,6,12^ | Mitra et al. 2015  Muzny et al. 2018  Chao et al. 2021 | |

1 Filardo, S. *et al.* Selected Immunological Mediators and Cervical Microbial Signatures in Women with Chlamydia trachomatis Infection. *mSystems* **4**, doi:10.1128/mSystems.00094-19 (2019).

2 Usyk, M. *et al.* Cervicovaginal microbiome and natural history of HPV in a longitudinal study. *PLoS Pathog* **16**, e1008376, doi:10.1371/journal.ppat.1008376 (2020).

3 Chao, X. *et al.* The role of the vaginal microbiome in distinguishing female chronic pelvic pain caused by endometriosis/adenomyosis. *Ann Transl Med* **9**, 771, doi:10.21037/atm-20-4586 (2021).

4 McClelland, R. S. *et al.* Evaluation of the association between the concentrations of key vaginal bacteria and the increased risk of HIV acquisition in African women from five cohorts: a nested case-control study. *Lancet Infect Dis* **18**, 554-564, doi:10.1016/s1473-3099(18)30058-6 (2018).

5 Romero, R. *et al.* The composition and stability of the vaginal microbiota of normal pregnant women is different from that of non-pregnant women. *Microbiome* **2**, 4, doi:10.1186/2049-2618-2-4 (2014).

6 Mitra, A. *et al.* Cervical intraepithelial neoplasia disease progression is associated with increased vaginal microbiome diversity. *Sci Rep* **5**, 16865, doi:10.1038/srep16865 (2015).

7 Alioua, S. *et al.* Diversity of Vaginal Lactic Acid Bacterial Microbiota in 15 Algerian Pregnant Women with and without Bacterial Vaginosis by using Culture Independent Method. *J Clin Diagn Res* **10**, Dc23-dc27, doi:10.7860/jcdr/2016/21621.8546 (2016).

8 Tabatabaei, N. *et al.* Vaginal microbiome in early pregnancy and subsequent risk of spontaneous preterm birth: a case-control study. *Bjog* **126**, 349-358, doi:10.1111/1471-0528.15299 (2019).

9 Shrestha, E. *et al.* Profiling the Urinary Microbiome in Men with Positive versus Negative Biopsies for Prostate Cancer. *J Urol* **199**, 161-171, doi:10.1016/j.juro.2017.08.001 (2018).

10 Si, J., You, H. J., Yu, J., Sung, J. & Ko, G. Prevotella as a Hub for Vaginal Microbiota under the Influence of Host Genetics and Their Association with Obesity. *Cell Host Microbe* **21**, 97-105, doi:10.1016/j.chom.2016.11.010 (2017).

11 Dareng, E. O. *et al.* Prevalent high-risk HPV infection and vaginal microbiota in Nigerian women. *Epidemiol Infect* **144**, 123-137, doi:10.1017/S0950268815000965 (2016).

12 Muzny, C. A. *et al.* Identification of Key Bacteria Involved in the Induction of Incident Bacterial Vaginosis: A Prospective Study. *J Infect Dis* **218**, 966-978, doi:10.1093/infdis/jiy243 (2018).

13 Dols, J. A. *et al.* Microarray-based identification of clinically relevant vaginal bacteria in relation to bacterial vaginosis. *Am J Obstet Gynecol* **204**, 305.e301-307, doi:10.1016/j.ajog.2010.11.012 (2011).

14 Onderdonk, A. B., Delaney, M. L. & Fichorova, R. N. The Human Microbiome during Bacterial Vaginosis. *Clin Microbiol Rev* **29**, 223-238, doi:10.1128/CMR.00075-15 (2016).

15 Borgdorff, H. *et al.* Lactobacillus-dominated cervicovaginal microbiota associated with reduced HIV/STI prevalence and genital HIV viral load in African women. *ISME J* **8**, 1781-1793, doi:10.1038/ismej.2014.26 (2014).

16 Huang, X. *et al.* Cervicovaginal microbiota composition correlates with the acquisition of high-risk human papillomavirus types. *Int J Cancer* **143**, 621-634, doi:10.1002/ijc.31342 (2018).

17 Fettweis, J. M. *et al.* The vaginal microbiome and preterm birth. *Nat Med* **25**, 1012-1021, doi:10.1038/s41591-019-0450-2 (2019).

18 Anahtar, M. N., Gootenberg, D. B., Mitchell, C. M. & Kwon, D. S. Cervicovaginal Microbiota and Reproductive Health: The Virtue of Simplicity. *Cell Host Microbe* **23**, 159-168, doi:10.1016/j.chom.2018.01.013 (2018).

19 Oh, H. Y. *et al.* The association of uterine cervical microbiota with an increased risk for cervical intraepithelial neoplasia in Korea. *Clin Microbiol Infect* **21**, 674 e671-679, doi:10.1016/j.cmi.2015.02.026 (2015).

20 Godoy-Vitorino, F. *et al.* Cervicovaginal Fungi and Bacteria Associated With Cervical Intraepithelial Neoplasia and High-Risk Human Papillomavirus Infections in a Hispanic Population. *Front Microbiol* **9**, 2533, doi:10.3389/fmicb.2018.02533 (2018).

21 Anahtar, M. N. *et al.* Cervicovaginal bacteria are a major modulator of host inflammatory responses in the female genital tract. *Immunity* **42**, 965-976, doi:10.1016/j.immuni.2015.04.019 (2015).

22 Ling, Z. *et al.* Molecular analysis of the diversity of vaginal microbiota associated with bacterial vaginosis. *BMC Genomics* **11**, 488, doi:10.1186/1471-2164-11-488 (2010).

23 Cox, R. A. & Slack, M. P. Clinical and microbiological features of Haemophilus influenzae vulvovaginitis in young girls. *J Clin Pathol* **55**, 961-964, doi:10.1136/jcp.55.12.961 (2002).

24 Gajer, P. *et al.* Temporal dynamics of the human vaginal microbiota. *Sci Transl Med* **4**, 132ra152, doi:10.1126/scitranslmed.3003605 (2012).

25 Srinivasan, S. *et al.* Megasphaera lornae sp. nov., Megasphaera hutchinsoni sp. nov., and Megasphaera vaginalis sp. nov.: novel bacteria isolated from the female genital tract. *Int J Syst Evol Microbiol* **71**, doi:10.1099/ijsem.0.004702 (2019).

26 Diop, K. *et al.* Characterization of a novel Gram-stain-positive anaerobic coccus isolated from the female genital tract: Genome sequence and description of Murdochiella vaginalis sp. nov. *Microbiologyopen* **7**, e00570, doi:10.1002/mbo3.570 (2018).

27 Margarita, V. *et al.* Two Different Species of Mycoplasma Endosymbionts Can Influence Trichomonas vaginalis Pathophysiology. *mBio* **13**, e0091822, doi:10.1128/mbio.00918-22 (2022).

28 Lithgow, K. V. *et al.* Protease activities of vaginal Porphyromonas species disrupt coagulation and extracellular matrix in the cervicovaginal niche. *NPJ Biofilms Microbiomes* **8**, 8, doi:10.1038/s41522-022-00270-7 (2022).

29 Mitra, A. *et al.* The vaginal microbiota associates with the regression of untreated cervical intraepithelial neoplasia 2 lesions. *Nat Commun* **11**, 1999, doi:10.1038/s41467-020-15856-y (2020).

30 Schaffer, J. N. & Pearson, M. M. Proteus mirabilis and Urinary Tract Infections. *Microbiol Spectr* **3**, doi:10.1128/microbiolspec.UTI-0017-2013 (2015).

31 Vornhagen, J. *et al.* Group B streptococcus exploits vaginal epithelial exfoliation for ascending infection. *J Clin Invest* **128**, 1985-1999, doi:10.1172/jci97043 (2018).
